# Histone H2A monoubiquitination marks are targeted to specific sites by cohesin subunits in *Arabidopsis*

**DOI:** 10.1038/s41467-023-36788-3

**Published:** 2023-03-03

**Authors:** Yu Zhang, Min Ma, Meng Liu, Aiqing Sun, Xiaoyun Zheng, Kunpeng Liu, Chunmei Yin, Chuanshun Li, Cizhong Jiang, Xiaoyu Tu, Yuda Fang

**Affiliations:** 1 Joint Center for Single Cell Biology, School of Agriculture and Biology, Shanghai Jiao Tong University, 200240 Shanghai, China; 2 Key Laboratory of Spine and Spinal Cord Injury Repair and Regeneration of Ministry of Education, Orthopaedic Department of Tongji Hospital, School of Life Sciences and Technology, Tongji University, 200065 Shanghai, China

**Keywords:** Plant molecular biology, Epigenomics, Epigenetics, Plant genetics

## Abstract

Histone H2A monoubiquitination (H2Aub1) functions as a conserved posttranslational modification in eukaryotes to maintain gene expression and guarantee cellular identity. *Arabidopsis* H2Aub1 is catalyzed by the core components AtRING1s and AtBMI1s of polycomb repressive complex 1 (PRC1). Because PRC1 components lack known DNA binding domains, it is unclear how H2Aub1 is established at specific genomic locations. Here, we show that the *Arabidopsis* cohesin subunits AtSYN4 and AtSCC3 interact with each other, and AtSCC3 binds to AtBMI1s. H2Aub1 levels are reduced in *atsyn4* mutant or *AtSCC3* artificial microRNA knockdown plants. ChIP-seq assays indicate that most binding events of AtSYN4 and AtSCC3 are associated with H2Aub1 along the genome where transcription is activated independently of H3K27me3. Finally, we show that AtSYN4 binds directly to the G-box motif and directs H2Aub1 to these sites. Our study thus reveals a mechanism for cohesin-mediated recruitment of AtBMI1s to specific genomic loci to mediate H2Aub1.

## Abstract (web summary)

How histone H2A monoubiquitination (H2Aub1) is established at specific genomic locations remains unclear. Here, the authors report that Arabidopsis cohesin subunits SCC3 and SYN4 are involved in H2Aub1 through their direct or indirect interaction with BMI1A/B/C subunits of PRC1, the E3 ligases in PRC1 for H2Aub1.

## Introduction

The nucleosome is the basic building block of chromatin and is composed of 146 base pairs (bp) of DNA wrapped around a histone octamer (H2A–H2B and H3–H4 dimers). The arrays of nucleosomes are connected by a shorter stretch of linker DNA and linker histone H1^1,2^. Histone tails (mostly within N-terminals) undergo various posttranslational modifications (PTMs), such as methylation, acetylation, phosphorylation, ubiquitination, and SUMOylation^3^. These PTMs impact genome functions by altering chromatin structure which plays critical roles in DNA-dependent processes, such as DNA replication, transcription, and damage repair^3^.

Histone ubiquitination mostly occurs in H2A and H2B, and may also target H1, H3, and H4^4^. H2A monoubiquitination (H2Aub1) occurs on K121 in *Arabidopsis*, K119 in vertebrates, or K118 in *Drosophila*^5^. In *Arabidopsis*, H2Aub1 is catalyzed by five RING-finger proteins (AtRING1A, AtRING1B, AtBMI1A, AtBMI1B, and AtBMI1C) in polycomb repressive complex 1 (PRC1)^5^. AtRING1A/B or AtBMI1A/B/C inactivation causes a dramatic reduction of the global level of H2Aub1 deposition and pleiotropic phenotypes related to *Arabidopsis* growth and stress responses^5,6^. Most recently, a study has reported that embryophytes have BMI1-like truncated genes (BMI1-L) that might compete with BMI1^7^.

Similar to mammalian systems, the prevailing model in which H3K27me3 deposition by PRC2 leads to the recruitment of PRC1 and subsequent deposition of H2Aub1 has once been accepted for the recruitment of Polycomb group (PcG) complexes to specific genomic loci in *Arabidopsis*^7,8^. This model explained that H2Aub1 is usually associated with the H3K27me3 mark and plays a repressive role in gene transcription^5,9^. However, recent studies have shown that the functions of PRC1/2 are complex^7^. H2Aub1 is essential for H3K27me3 and PRC1-mediated transcriptional regulation in *Marchantia polymorpha*^10^. PRC1 activity is required for the H3K27me3 modification of seed maturation genes in *Arabidopsis*^11^. Moreover, *Arabidopsis* H3K27me3 enrichment covers gene bodies whereas H2Aub1 is enriched at the regions surrounding the transcription start site (TSS)^12,13^. The ubiquitination-independent repression by PRC1 plays an important role in determining neuronal fate in mammals^14^. Nevertheless, PRC1 can associate with polycomb response elements (PREs) even without PRC2 participation in *Drosophila*^15,16^. EMBRYONIC FLOWER 1 (EMF1) and LIKE HETEROCHROMATIN PROTEIN 1 (LHP1) were once thought to be PRC1 subunits but were recently identified as PRC2-associated proteins^7,17^. EARLY BOLTING IN SHORT DAYS (EBS) and its homolog SHORT LIFE (SHL) were also defined as PRC2-associated proteins^18^, and PRC-associated coiled-coil protein plays a role in the maintenance of rice shoot apical meristem activity by regulating H3K27me3^19^. As PRC1 contains no DNA-binding domain, the molecular mechanism for recruiting PRC1 to the specific genomic loci remains unclear.

The cohesin complex is highly conserved in eukaryotes and consists of SISTER CHROMATID COHESION 3 (SCC3), the α-kleisin family protein, and structural maintenance of chromosome (SMC) proteins SMC1 and SMC3. Within cohesin, SMC1 and SMC3 proteins form a long-armed V-shaped heterodimer, and the α-kleisin subunit bridges the SMC dimer, forming a ring-like structure. SCC3 interacts with the α-kleisin subunit, and stabilizes the ring-like structure^20^. In *Arabidopsis*, homozygous T-DNA knockout mutants of *AtSMC1*, *AtSMC3*, and *AtSCC3* show developmental defects in embryo and endosperm, underlying early developmental arrest in the seed^21–24^. The *Arabidopsis* genome encodes single copies of *AtSMC1*, *AtSMC3*, and *AtSCC3*, but four α-kleisin genes (*AtSYN1–4*)^25,26^. AtSYN1 is a meiosis-specific cohesin subunit, and the homozygous *atsyn1* T-DNA insertion line is sterile in male and female gametophytes^25,27,28^. AtSYN3 is indispensable for meiotic recombination, and the null allele of AtSYN3 shows gametophyte lethality^26,29^. AtSYN2 and AtSYN4 play roles in DNA double-strand break (DSB) repair^30,31^. After recruitment to chromatin, cohesin is established and maintained in the genome by the activity of an acetyltransferase (establishment of cohesin 1/Chromosome transmission fidelity 7, Eco1/Ctf7). Most of the homozygous *atctf7* mutant seeds show embryo arrest during their early development. Only a small number of *atctf7* plants can survive, but exhibit major defects in vegetative growth and development, and are completely sterile^32^. Initially, cohesin was found to function as a ‘molecular glue’ to cohere sister chromatids. In addition to the well-illustrated function of cohesin in mitosis and meiosis^33^, growing evidence has indicated a pivotal role of cohesin in modulating the three-dimensional (3D) genome organization and transcriptional regulation^34^. In animals, cohesin and CCCTC­binding factor (CTCF) are essential for forming topologically associating domains. The dynamic interaction of cohesin with its chromatin binding site builds a bridge between enhancers/insulators and promoters, thereby regulating gene expression^34^. In humans, the cohesin–NIPBL complex mediates chromatin loop extrusion by a ‘swing and clamp’ mechanism^35^. In *Drosophila*, PRC1 subunits and cohesin subunits SMC1/3 can be co-purified^36^. However, a functional link between cohesin and PRC1 has not been established, and the potential coordination of cohesin and H2Aub1 remains to be illustrated.

In this work, we show that AtSYN4 interacts with AtSCC3 which binds to AtBMIs. In addition, the cohesin subunits AtSYN4 and AtSCC3 are functionally related to catalytic subunits of PRC1, AtBMI1A/B/C. Chromatin immunoprecipitation followed by sequencing (ChIP-seq) assays indicate that most binding events of AtSYN4 and AtSCC3 are associated with H2Aub1 along the genome. Moreover, AtSYN4 directly binds to the genomic loci enriched with the G-box motif of DNA and associates with AtBMI1A/B/C through AtSCC3, thus affecting H2Aub1 levels at these sites. Together, these results reveal the role of cohesin subunits in the recruitment of AtBMIs to certain genomic loci to mediate H2Aub1 in these specific locations.

## Results

### Cohesin subunit AtSCC3 interacts with AtBMI1A/B/C

**Fig. 1:**
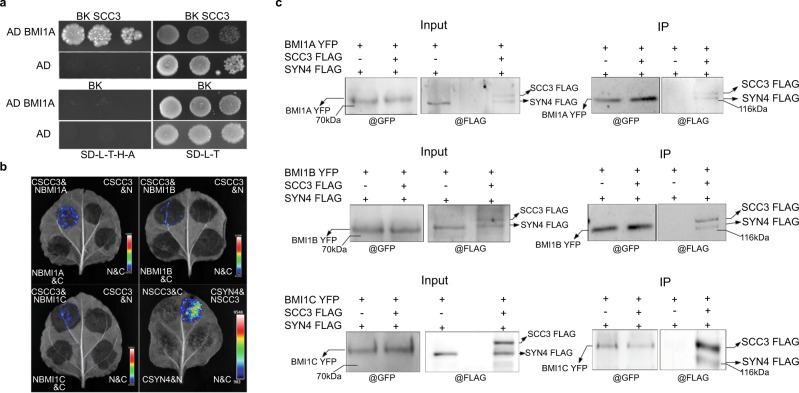
AtSCC3 interacts with AtSYN4 and AtBMI1A/B/C. **a** Yeast two-hybrid assays show that AtSCC3 interacts with AtBMI1A. **b** LUC complementation imaging assays show that AtSCC3 interacts with AtBMI1A/B/C and AtSYN4. CSCC3 or NSCC3 represents the AtSCC3 fused with the C-terminal or N-terminal of LUC, respectively. NBMI1A/B/C represents AtBMI1A/B/C fused with the N-terminal of LUC. CSYN4 represents AtSYN4 fused with the C-terminal of LUC. **c** Co-IP experiments verified that AtSYN4 can interact with AtBMI1A/B/C only when AtSCC3 is expressed. The experiment was repeated independently three times with similar results. The unprocessed scans of the blot images are provided as a Source Data file.

To explore the mechanism for H2Aub1 targeting, we screened for AtBMI1A partners by yeast two-hybrid assays and identified AtSCC3, a subunit of cohesin (Fig. 1a). Luciferase (LUC) complementation assays showed that AtSCC3 interacts with AtBMI1A and its homologs, AtBMI1B and AtBMI1C (Fig. 1b). Interestingly, LUC complementation assay indicated that AtSYN4, but not AtSYN1–3, interacts with AtSCC3 when transiently expressed in tobacco leaf cells (Fig. 1b and Supplementary Fig. 1a). Coimmunoprecipitation (Co-IP) further confirmed that AtSYN4 directly interacts with AtSCC3 (Supplementary Fig. 1b). We have not detected a direct interaction between AtSYN4 and AtBMI1A/B/C through luciferase complementation assay. Given that AtSCC3 can interact with AtSYN4 and AtBMIs (Fig. 1a, b and Supplementary Fig. 1b), we speculated that AtSCC3 might act as a bridge that can connect AtSYN4 with AtBMI1s. To test this hypothesis, we performed an in vivo Co-IP assay. When AtBMI1A-YFP, AtSYN4-FLAG, and AtSCC3-FLAG were co-expressed in tobacco leaves, AtSCC3-FLAG and AtSYN4-FLAG could be pulled down by AtBMI1A-YFP. In contrast, when AtBMI1A-YFP and AtSYN4-FLAG were co-expressed in tobacco leaves without AtSCC3-FLAG, AtSYN4-FLAG could not be pulled down by AtBMI1A-YFP. Similar results were obtained from AtBMI1B-YFP and AtBMI1C-YFP (Fig. 1c; Supplementary Fig. 1c).

AtBMI1A/B/C can bind AtRING1A or AtRING1B^37^. Therefore, we examined the potential interactions between AtSCC3 or AtSYN4 and AtRING1A or AtRING1B. We found that AtRING1A interacts with AtSCC3 and AtSYN4, and the interaction between AtRING1A and AtSCC3 is much stronger than that between AtRING1A and AtSYN4 (Supplementary Fig. 1d).

### AtSCC3 and AtSYN4 functionally correlate with AtBMI1A/B/C

*Arabidopsis* has a single copy of the *AtSCC3* gene and its homozygous full loss-of-function alleles are embryonic lethal^24^. Therefore, we knocked down *AtSCC3* by using artificial microRNAs (amiRNAs) with the miR319a precursor as a backbone. We obtained two RNA interference (RNAi) lines, *SCC3*RNAi-1 and *SCC3*RNAi-2, with the transcript level of *AtSCC3* decreasing by approximately 1/3 and 2/3, respectively (Supplementary Fig. 2a). In addition to *AtSCC3* knockdown lines, we also used the *atctf7* mutant, in which the cohesin complex cannot be stably maintained on DNA^38^, and *atctf7* seedlings display severe dwarf and major defects in vegetative growth and development (Supplementary Fig. 2b).

**Fig. 2:**
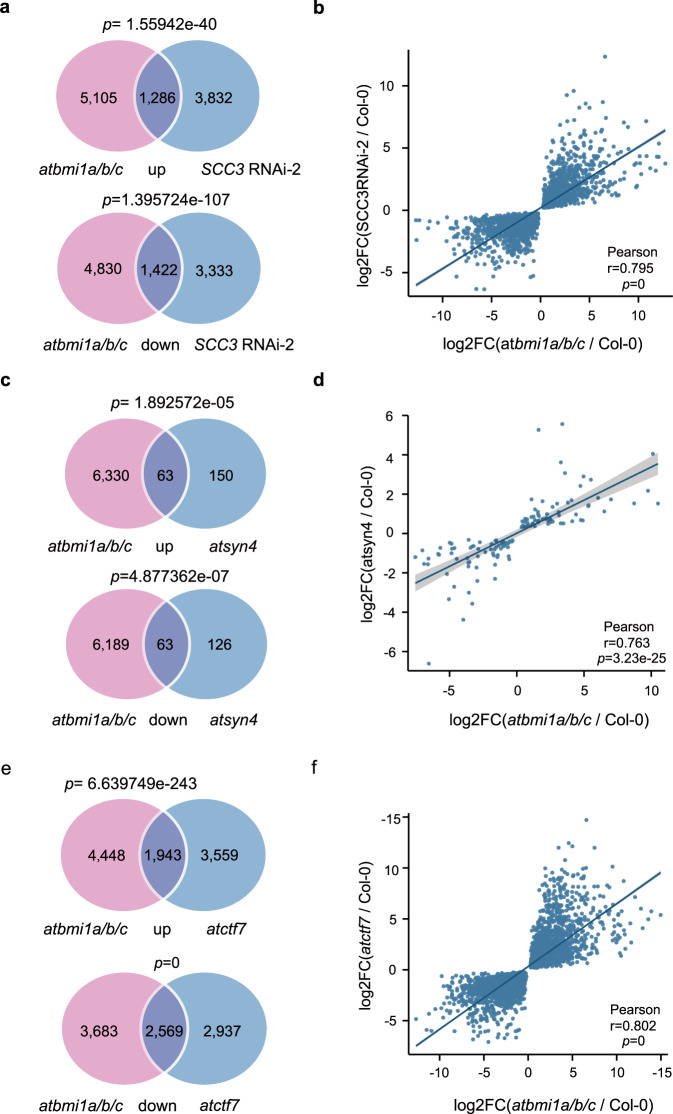
Transcriptome comparisons between atbmi1a/b/c and atsyn4, atctf7, or SCC3RNAi-2 plants. **a** Venn diagram showing overlaps of the up- or down-regulated genes between *SCC3*RNAi-2 and *atbmi1a/b/c* mutants. Significance was examined by the hypergeometric test. **b** Scatter plots showing positive correlations of up- and down-regulated genes between *SCC3*RNAi-2 and *atbmi1a/b/c* mutants. Statistical analysis was performed with two-tailed Student’s *t*-tests. |log_2_fold change | > 0 and *p* adjusted <0.05. The values of log2FC(*atbmi1a/b/c*/Col-0) and log2FC(*SCC3*RNAi-2/Col-0) are provided as a Source Data file. **c** Venn diagrams showing the overlaps of up- or down-regulated genes between *atsyn4* and *atbmi1a/b/c* mutants. Significance was examined by the hypergeometric test. **d** Scatter plots showing positive correlations of up- and down-regulated genes between *atsyn4* and *atbmi1a/b/c* mutants. Statistical analysis was performed with two-tailed Student’s *t*-tests. |log_2_fold change | > 0 and *p* adjusted < 0.05. The values of log2FC(*atbmi1a/b/c*/Col-0) and log2FC(*atsyn4*/Col-0) are provided as a Source Data file. **e** Venn diagrams showing the overlaps of the up- or down-regulated genes between *atctf7* and *atbmi1a/b/c* mutants. Significance was examined by the hypergeometric test. **f** Scatter plots showing positive correlations of the up- and down-regulated genes between *atctf7* and *atbmi1a/b/c* mutants. Statistical analysis was performed with two-tailed Student’s *t*-tests. |log_2_fold change | > 0 and *p* adjusted < 0.05. The values of log2FC(*atbmi1a/b/c*/Col-0) and log2FC(*atctf7*/Col-0) are provided as a Source Data file.

*Atsyn4*, *SCC3*RNAi-1, *SCC3*RNAi-2, and *atbmi1a/b* mutant plants all exhibit phenotypes of delayed flowering and serrated rosette leaves (Supplementary Fig. 2c–e). The phenocopies among *atsyn4*, *SCC3*RNAi-1, *SCC3*RNAi-2, and *atbmi1a/b* mutant lines and the physical interactions among AtSCC3, AtSYN4, and AtBMIs implicated the potential functional connections among these proteins. We then performed RNA sequencing (RNA-seq) to quantify transcripts in 7-day-old seedlings of Col-0, *atsyn4*, *SCC3RNAi-2*, and *atctf7*. The quality-control metrics of RNA-seq data are shown in Supplementary Fig. 3a, b. We found that the differentially expressed genes (DEGs) between *SCC3RNAi-2* and *atbmi1a/b/c*^12^ significantly overlapped and were positively correlated (Fig. 2a, b, Supplementary Data 1, 2). In the *atsyn4* mutant, 402 DEGs overlapped significantly, and there existed a strong positive correlation of log_2_-transformed fold changes from RNA-seq analysis between *atsyn4* and *atbmi1a/b/c* mutants (Fig. 2c, d, Supplementary Data 2, 3). As *AtSYN2* and *AtSYN4* play a role in DNA DSB repair^30,31^, we generated an *atsyn2atsyn4* double mutant (Supplementary Fig. 4a) and performed RNA-seq analysis of the mutant. Compared to *atsyn4*, with only 402 DEGs, the *atsyn2atsyn4* double mutant has 3314 DEGs which overlap with those in *atbmi1a/b/c* (Supplementary Fig. 4b, Supplementary Data 2, 4). However, up- or down-regulated genes between *atsyn2atsyn4* and *atbmi1a/b/c* are not significantly correlated (Supplementary Fig. 4c). These results implicated that AtSYN4 plays a significant role in the AtBMI-mediated pathway, and *AtSYN2* might be involved in this process to some degree upon the complete depletion of *AtSYN4*. Comparison of *atctf7* and *atbmi1a/b/c* transcriptome data showed that genes with the same trend of disordered expression are also apparently intersected, and positively correlated (Fig. 2e, f, Supplementary Data 2, 5).

Given that AtSCC3 interacts with AtSYN4 and AtBMI1A/B/C which are associated with H2Aub1, we examined the global H2Aub1 levels in *atsyn4*, *SCC3*RNAi-1, *SCC3*RNAi-2, and *atctf7* mutants. Western blots showed that the global levels of H2Aub1 in *SCC3*RNAi-2 and *atctf7* mutant plants are much lower than that in Col-0 (Supplementary Fig. 5a). Consistent with the weaker phenotypes of *atsyn4* and *SCC3*RNAi-1 lines than *SCC3*RNAi-2 line (Supplementary Figs. 2e and  5b), a milder decrease of the global H2Aub1 level in *atsyn4* and *SCC3*RNAi-1 lines than *SCC3*RNAi-2 was detected (Supplementary Fig. 5a).

### AtSYN4/AtSCC3 and H2Aub1 tend to flank TSS closely

To address genomic relationships among AtSYN4, AtSCC3, and H2Aub1, we first detected the genome-wide occupancy of AtSYN4 and AtSCC3 using ChIP-seq. The quality-control metrics of ChIP-seq experiments are shown in Supplementary Fig. 6a, b. We used amino acids 898–1098 of AtSCC3 as an antigen to generate an AtSCC3-specific antibody named CSCC3 (Supplementary Fig. 7a) for AtSCC3 ChIP-seq (Supplementary Data 6). A green fluorescent protein (GFP) antibody and the *pSYN4*-*SYN4-YFP*/*atsyn4* transgenic line (Supplementary Fig. 7b) were used for AtSYN4 ChIP-seq (Supplementary Data 7).

**Fig. 3:**
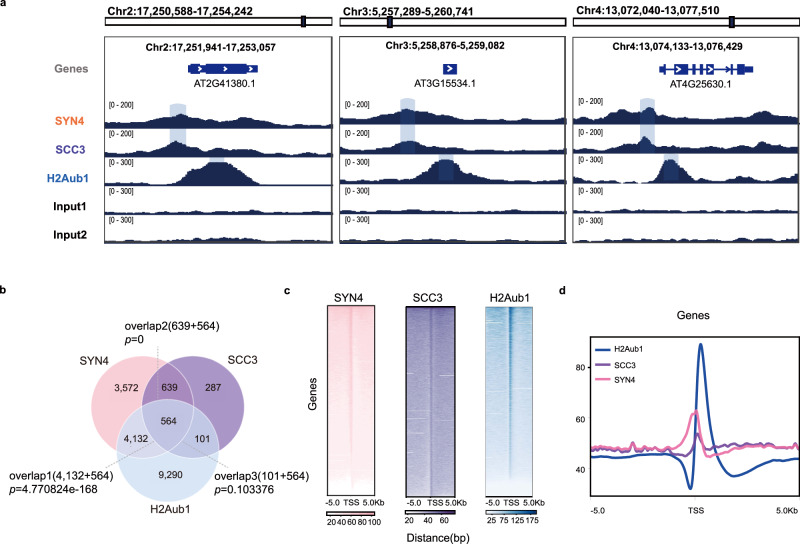
Co-occupied AtSYN4 and AtSCC3 locate adjacent to H2Aub1 sites across the TSS in the genome. **a** IGV screenshots of AtSYN4, AtSCC3, and H2Aub1 showing enrichments at the loci of selected genes. Three genes selected are *AT2G41380* (Chr2:17,251,941–17,253,057), *AT3G15534* (Chr3:5,258,876–5,259,082), and *AT4G25630* (Chr4:13,074,133–13,076,429). **b** Venn diagrams showing the overlaps of genes targeted by AtSYN4/AtSCC3 and marked by H2Aub1. Significance was examined by the hypergeometric test. **c** Signal density heatmaps across the ±5 kb of TSS in genes targeted by AtSYN4/AtSCC3 and marked by H2Aub1. **d** ChIP-seq enrichments across the ±5 kb of TSS in genes targeted by AtSYN4/AtSCC3 and marked by H2Aub1.

We then genome-widely compared AtSCC3 and AtSYN4 targeted genes to H2Aub1 occupancy^12^. By visualizing on Integrative Genomics Viewer (IGV), we observed that AtSYN4 and AtSCC3 peaks tend to locate slightly upstream of the TSS, and the peaks of H2Aub1 locate slightly downstream of the TSS (Fig. 3a). In addition, we found that a large proportion of genes (~76%, 1203 of 1591) bound by AtSCC3 showed significant enrichment of AtSYN4. About 53% (4696 of 8907) and 42% (665 of 1591) of genes occupied by AtSYN4 and AtSCC3, respectively, are H2Aub1-marked genes (Fig. 3b). We then compared AtSYN4, AtSCC3, and H2Aub1 enrichment patterns across the ±5 kb flanking TSS. The heatmap and signal density plots across the ±5 kb flanking TSS suggested that AtSYN4 colocalizes largely with AtSCC3 along the genome, and the AtSCC3/AtSYN4 peaks locate at ~100 bp upstream of the TSS (Fig. 3c, d, Supplementary Data 6, 7), and H2Aub1 ChIP-seq peaks are enriched at ~250 bp downstream of the TSS (Fig. 3c, d).

**Fig. 4:**
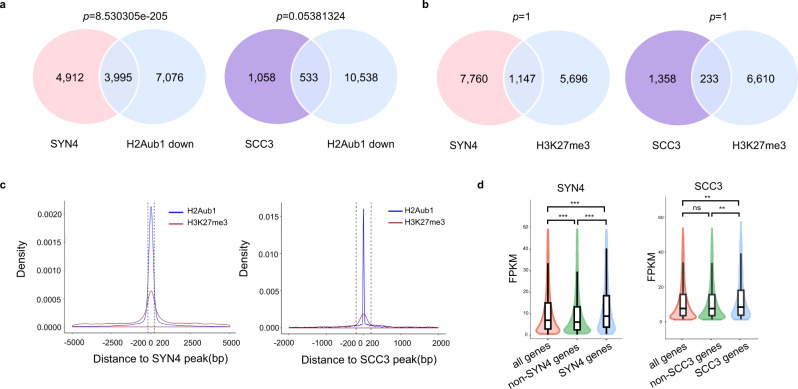
AtSYN4 and AtSCC3 are associated functionally with H2Aub1 and active transcription. **a** Venn diagram showing the overlaps between the genes with downregulated H2Aub1 levels in *atbmi1a/b/c* and the genes targeted by AtSYN4 (left) or AtSCC3 (right) in Col-0. Significance was examined by the hypergeometric test. **b** Venn diagrams showing no significant intersection between genes marked by H3K27me3 and those targeted by AtSYN4 (left) or AtSCC3 (right) in Col-0. Significance was tested using a hypergeometric test. **c** Density plots displaying the distances of H2Aub1 and H3K27me3 to the center of AtSYN4 peak (left) or AtSCC3 peak (right). The dotted line represents the average distance to AtSYN4 or AtSCC3 peak. **d** Comparisons of the transcription levels between AtSYN4- or AtSCC3-targeted genes and not targeted genes. The *p*-value was calculated by the one-sided Wilcoxon test, *p*-value: “***“ < 0.001, “**“ < 0.01, “ns” not significant. Data are mean ± SD of three biological repeats. The properties of the box plots are defined as follows; minima: lower whisker = smallest observation greater than or equal to lower hinge−1.5*IQR (IQR = interquartile range: the difference between the 75th and 25th percentiles), box lower hinge = 25% quantile; box middle = median, 50% quantile; box upper hinge = 75% quantile; maxima: upper whisker = largest observation less than or equal to upper hinge + 1.5*IQR. The transcription levels of AtSYN4- or AtSCC3-targeted genes and not targeted genes are provided as a Source Data file.

Next, we analyzed the genes with their H2Aub1 levels downregulated by *AtBMI* mutations. We found that the genes with decreased H2Aub1 levels in the *atbmi1a/b/c* triple mutant overlap significantly with AtSYN4- or AtSCC3-targeted genes in Col-0 (Fig. 4a), in contrast to the rare gene sharing between AtSYN4/AtSCC3 and H3K27me3 targets (Fig. 4b). In addition, H2Aub1 peaks were found closer to AtSYN4 or AtSCC3 binding sites than regions associated with the H3K27me3 signature (Fig. 4c). To examine the transcriptional states of AtSYN4 and AtSCC3 target genes, we quantified the relative transcript levels of their target genes by RNA-seq on 7-day-old wild-type seedlings. We found that the transcript levels of AtSYN4 or AtSCC3 targeted genes are significantly higher than genes nontargeted by AtSYN4 or AtSCC3, respectively (Fig. 4d), indicating that genes associated with AtSYN4/AtSCC3 are more transcriptionally active^12^.

Based on combined AtSYN4/AtSCC3 ChIP-seq and *atsyn4/AtSCC3*RNAi-1 transcriptome analysis, we found that the DEGs identified in *atsyn4* and *AtSCC3*RNAi-1 are significantly related to AtSYN4 and AtSCC3 target genes marked by H2Aub1, respectively (Supplementary Fig. 8a and 8b). Gene Ontology (GO) analysis indicated that the representative categories based on biological function include responses to wounding, chitin, and water deprivation (Supplementary Fig. 8c, 8d).

### AtSYN4 and AtSCC3 affect H2Aub1 levels neighboring G-box-containing DNA

**Fig. 5:**
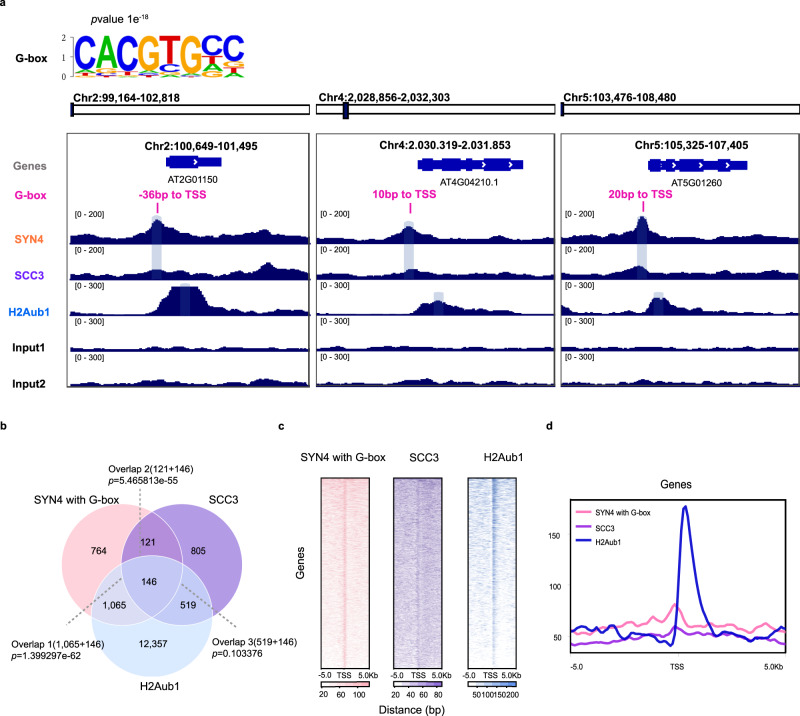
AtSYN4 affects H2Aub1 in G-box-containing DNA sequences. **a** The G-box motif feature in AtSYN4, AtSCC3, and H2Aub1 binding sites. IGV screenshots of AtSYN4, AtSCC3, and H2Aub1 showing AtSYN4/AtSCC3 peaks occupy the G-box sites of selected genes and locate close to H2Aub1 around TSSs. Three genes selected are *AT2G01150* (Chr100,649–101,495), *AT4G04210* (Chr4:2,030,319–2,031,853) and *AT5G01260* (Chr5:105,325–107,405). **b** Venn diagrams showing overlaps of the genes targeted by AtSYN4 at G-box, AtSCC3, and marked by H2Aub1. Significance was examined by the hypergeometric test. **c** Signal density heatmaps across the ±5 kb flanking TSS in the G-box-containing genes targeted by AtSYN4, AtSCC3, and H2Aub1. **d** ChIP-seq enrichments across the ±5 kb flanking TSS in the G-box-containing genes targeted by AtSYN4, AtSCC3, and H2Aub1.

How AtBMI1A/B/C finds the target sites and mediates H2Aub1 in a DNA sequence-specific manner is not clear. We speculated that AtSYN4 and AtSCC3 could facilitate AtBMI1A/B/C binding to a specific DNA sequences. To test the hypothesis, we identified the G-box motif (CACGTG) in the co-occupied loci of AtSYN4, AtSCC3, and H2Aub1 (Fig. 5a).

The IGV screenshots of the selected genes showed that AtSYN4 peaks closely locate the G-box sites and H2Aub1 peaks are adjacent to these G-box sites (Fig. 5a). In addition, more than half of the genes (~58%, 1211 of 2106) with the G-box bounded by AtSYN4 are marked by H2Aub1 (Fig. 5b), and about 35% (564 of 1591) of the genes with the G-box bounded by AtSCC3 are H2Aub1-marked. Moreover, we plotted AtSYN4, AtSCC3, and H2Aub1 enrichment patterns across the ±5 kb flanking TSS of genes targeted by AtSYN4 with the G-box element (Fig. 5c, d). The heatmap and signal density plots centered on TSS suggested that the AtSYN4 peaks with the G-box element had similar chromatin signatures with AtSCC3 and H2Aub1 across the TSS (Figs. 3c, d and 5c, d).

**Fig. 6:**
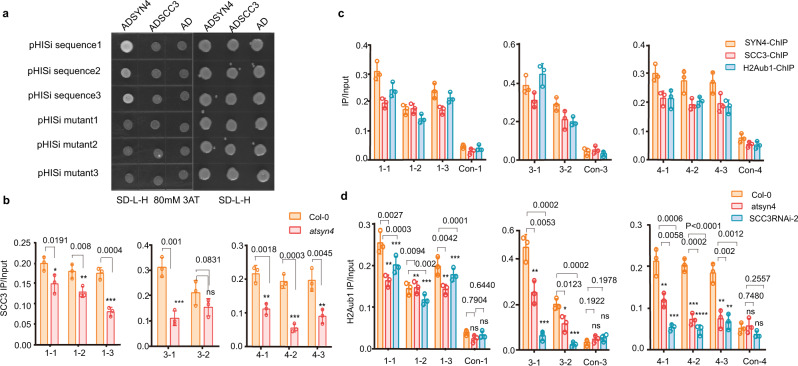
AtSYN4 can bind the G-box-containing DNA directly. **a** Yeast one-hybrid assay shows that AtSYN4, but not AtSCC3, binds to the G-box motif-containing DNA. Sequence 1, sequence 2, and sequence 3 were 50 bp G-box (CACGTG) containing DNA selected from the promoter regions of *AT1G64385* (chr1:23,899,109–23,899,158), *AT3G23440* (chr3: 8,404,698–8,404,747), and *AT4G16380* (chr4: 9,254,461–9,254,510), respectively. Mutant 1, mutant 2, and mutant 3 were corresponding sequences with CACGTG mutated into GGGGGG and used as the control. Yeast transformants were grown on synthetic dropout (SD-Leu-His) plates with 3-AT (80 mM) for 4 days. **b** ChIP-qPCRs show that AtSCC3 occupancy on DNA was severely reduced in *atsyn4* mutant. Data are mean ± SD of three biological repeats. Statistical analysis was performed with one-tailed Student’s *t* tests; *p*-value: “***“ < 0.001, “**“ < 0.01, “ns” not significant. **c** ChIP-qPCRs verify the enrichments of AtSCC3, AtSYN4, and H2Aub1 in the selected sites. Data are mean ± SD of three biological repeats. **d** ChIP-qPCRs show that H2Aub1 occupancy on DNA was severely reduced in *atsyn4* and *SCC3*RANi-2 lines. Data are mean ± SD of three biological repeats. Statistical analysis was performed with one-tailed Student’s *t* tests; *p*-value: “***“ < 0.001, “**“ < 0.01, “ns” not significant. The exact *p* values are noted and the raw data of the bar graphs are provided as a Source Data file.

**Fig. 7:**
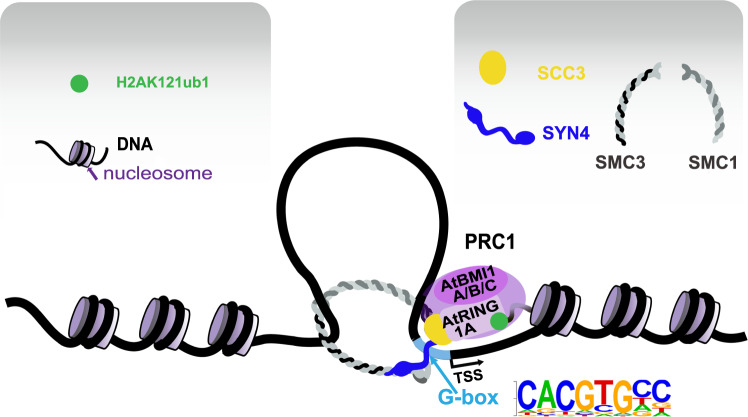
A proposed model for the mechanism elucidating the specific targeting of H2Aub1 to G-box-containing loci through cohesin. The cohesin subunit AtSYN4 binds to the G-box motif upstream of the TSS, where AtSCC3 interacts with AtSYN4 and AtBMI1A/B/C subunits in PRC1, thereby recruiting these enzymes to the G-box-containing loci and facilitating the monoubiquitination of H2A in the first nucleosome downstream of the TSS.

We then employed yeast one-hybrid (Y1H) assays to study the specific recognition between AtSYN4/AtSCC3 and the selected three DNA sequences containing the G-box in the AtSYN4, AtSCC3, and H2Aub1 co-occupied loci. The results showed that AtSYN4 could bind the G-box containing DNA directly, blocked by the mutations in the G-box (Fig. 6a). We found that the AtSCC3-occupied DNA in this G-box containing loci decreases in the *atsyn4* mutant (Fig. 6b). We further performed ChIP-qPCRs and confirmed that AtSYN4, AtSCC3, and H2Aub1 occupy DNA fragments containing the G-box motif (Fig. 6c). In addition, we found that H2Aub1 levels decrease at these G-box-containing loci in *atsyn4* and *SCC3*RNAi-2 plants (Fig. 6d). Altogether, these findings supported a model in which AtSYN4 directly binds to the G-box containing DNA, and AtSCC3 functions as a linker between AtSYN4 and AtBEMI1A/B/C, which in turn recruits AtBMI1A/B/C to this G-box containing loci to mediate H2Aub1 on nucleosomes at these specific genomic sites (Fig. 7).

## Discussion

Developmental transitions and cell fate decisions are key processes of the *Arabidopsis* lifecycle and require specific and stable remodeling of gene expression patterns. PcG proteins are crucial epigenetic regulators of developmental transitions and cell fate decisions. PRC1 and PRC2 play a role in seed embryonic trait determinacy. Numerous embryo-characteristic genes are ectopically expressed in various *PRC1* and *PRC2* mutant seedlings, as reflected at a morphological level by indeterminate growth, resulting in the formation of callus-like structures^5,37,39,40^. Besides, PRC1 and PRC2 also function in shoot stem cell fate determinacy^5,9,41^. Similar to AtBMI1s^5,9,41^, we found AtSYN4 or AtSCC3 depletion can prolong the vegetative growth period, characterized by late flowering, implicating that cohesin-mediated H2Aub1 might play a role in the transition from vegetative growth to reproductive flowering in *Arabidopsis*.

H2Aub1 in *Arabidopsis* is catalyzed by PRC1 and acts as a pivotal regulator of chromatin-associated processes, including DNA replication, damage repair, and gene expression^15,42^. The traditional view is that H2Aub1 starts with H3K27me3 modification by PRC2. Then, PRC1 binds/reads H3K27me3 via its subunits and further catalyzes H2Aub1, which plays a repressive role in transcriptional regulation. Recent studies have come up with different models. For example, H2Aub1 may lead to the recruitment of PRC2. In other words, PRC1 works upstream of PRC2^11,43^. H2Aub1 marks are widespread in the *Arabidopsis* genome, not only colocalizing with H3K27me3 but also occupying a set of transcriptionally active genes devoid of H3K27me3^12,13^. However, how PRC1 is recruited to loci in an H3K27me3-independent manner and mediates downstream functions are not answered.

In this study, we found that cohesin subunits AtSCC3 and AtSYN4 are involved in H2Aub1 through their direct or indirect interaction with AtBMI1A/B/C, the E3 ligases in PRC1 for H2Aub1. By direct interaction between AtSCC3 and AtSYN4 which binds to genomic loci containing consensus G-box motifs, AtSCC3 recruits AtBMIs to these genomic loci to monoubiquitinate H2A. AtSCC3 and AtBMIs might be more dynamic than AtSYN4 at these sites as more than half of G-box-containing genes bounded by AtSYN4 are marked by H2Aub1, and fewer G-box-containing genes bounded by AtSCC3 are marked by H2Aub1 (Fig. 5b), supporting a ‘work and go’ model for these proteins, especially for AtSCC3 and AtBMI1A/B/C. Downregulation of AtSCC3 by RNAi or AtSYN4 mutation causes the global reduction of the H2Aub1 level. In addition, cohesin-associated H2Aub1 is preferentially deposited to transcriptionally active genes. Our results thus largely revealed the mechanism for H2Aub1 targeting and gene regulation through the cohesin-PRC1 module.

*Arabidopsis* genome encodes four α-kleisin genes, *AtSYN1-4*^25,26^. The functions of AtSYN1 (the orthologue of yeast Rec8) and AtSYN3 seem to be meiosis-specific^25–29^. In this study, a specific role of AtSYN4 in H2Aub1 is reported. AtSYN2 might be involved in the AtSYN4-mediated process to some degree upon the complete deletion of AtSYN4. The functions of *Arabidopsis* α-kleisin-like proteins AtSYN1-4 might be cell cycle, cell type, tissue, or development stage-specific; therefore, the functional divergence among AtSYN1-4 is of interest to be fully investigated in the future.

PREs are a series of DNA sequences that interact with PcG proteins^44^. PREs can be recognized by specific transcription factors to recruit PRC1 and subsequently cooperate with additional proteins through DNA–protein or protein–protein interactions^45–47^. Interestingly, PREs can exist long distances (thousands of base pairs) before the TSS^45,48–50^. If and how PRC1 plays a role in long-range regulation remains a mystery. Although the role of cohesin in 3D genome organization, including chromatin loop formation, was revealed in mammalian systems, our model raised a possibility regarding the role of PREs in the long-distance regulation of H2Aub1 through cohesin, which might mediate long-distance chromatin interaction and recruit catalytic subunits in PRC. In addition, we found that AtSYN4 and AtSCC3 are mostly located within promoter regions, typically the core promoter regions immediately upstream of the TSS, and that there is a shift of ~350 bp between H2Aub1 and these two cohesin subunits. This may be related to the occupancy of nucleosomes around the TSS as a ~140 bp nucleosome-free promoter region exists upstream of the TSS, and the sites with the highest occupancy of nucleosomes are located 100 bp downstream of the TSS^51,52^. Cohesin mediates promoter–enhancer or promoter–insulator interactions in animals^34^. Considering the conservation of cohesin structure and function, it is interesting to study whether plant cohesin mediates long-range chromatin interactions and long-distance H2Aub1. However, the plant has no homolog of the CTCF factor. It is still an open question if there are CTCF-like factors in plants that play a role in 3D chromatin organization together with cohesin, similar to animal systems.

## Methods

### Plant materials and growth conditions

*Arabidopsis thaliana* (ecotype Col-0), T-DNA insertion mutants *atsyn2* (SALK_044851), *atsyn4* (SALK_076116), *atctf7* (SALK_059500) were obtained from the ABRC stock center at Ohio State University. *Atsyn2* was crossed with *atsyn4* to obtain *atsyn2atsyn4* double mutant. *Atbmi1a/b* mutant^37^ was a gift from Prof. Lin Xu (CEMPS, SIPPE). All the mutants were confirmed by PCR with primers listed in Supplementary Data 8. Plant materials were grown at 21 °C on agar plates containing Murashige & Skoog (MS) medium supplemented with 1.5% sucrose and 0.8% agar^12^. For visualization of phenotypes, after stratification at 4 °C for 3 days, the seedlings were grown in soil at 21 °C in long-day conditions (16 h light and 8 h dark cycles). The days of flowering and rosette numbers were scored with three biological replicates.

### Yeast two-hybrid assay

Yeast two-hybrid interaction assays were performed according to the Yeast Maker Yeast Transformation System 2 User Manual (Clontech). The coding sequences of *AtSCC3* and *AtBMI1A* were subcloned into pGBKT7 or pGADT7, respectively. The constructs were then co-transformed into yeast (AH109). The yeast cells containing the bait and the prey constructs were grown on selective plates (SD-Leu-Trp-His-Ala and SD-Leu-Trp) for analysis. The results were tested after 3–7 days of growth at 30˚C. The primers used are listed in Supplementary Data 8. Values came from three biological replicates.

### Firefly luciferase (LUC) complementation imaging assay

For LUC complementation imaging assays^53^, *AtSYN4*, *AtSCC3*, *AtBMI1A/B/C*, and *AtRING1A* were fused to the N- or C-terminal fragment of *LUC* (N and C), respectively. The primers used are listed in Supplementary Data 8. The fused plasmids were introduced into *Agrobacterium tumefaciens* strain GV3101 by electroporation and then incubated in LB (with 50 mg/L kanamycin and 25 mg/L gentamycin) plate medium at 30 °C for 48–72 h to OD_600_ = 0.8. Then mix the corresponding *Agrobacterium tumefaciens* strains equally and then co-infiltrated into tobacco (*N. benthamiana*) leaves using an injection syringe. 48 h later, the infiltrated leaves were injected with 100 mM luciferin (Sango, dissolved in water), and the luciferase signals were detected by the PMCapture software (Version 1.00) of a Chemiluminescence Imaging System (Tanon 5500, Shanghai, China). Values came from three biological replicates.

### Co-Immunoprecipitation (Co-IP) assay

The coding sequences of *AtSCC3*, *AtSYN4*, *AtBMI1A, AtBMI1C*, *and AtBMI1B* were subcloned into *Eco*RI/*Sal*I-treated vector *pCambia1300-35S-N1-FLAG*, respectively. Primers used were shown in Supplementary Data 8. The plasmids containing *AtSCC3-FLAG*, *AtSYN4-FLAG*, *AtBMI1A-YFP*, *AtBMI1B-YFP,* or *AtBMI1C-YFP* expressing cassettes were introduced into *Agrobacterium tumefaciens* GV3101 by electroporation and then incubated in LB (with 50 mg/L kanamycin and 25 mg/L gentamycin) plate medium at 30 °C for 48–72 h to OD_600_ = 0.8. For co-expression of AtBMI1A/B/C-YFP and AtSYN4-FLAG with or without AtSCC3-FLAG, the corresponding *Agrobacterium tumefaciens* strains were mixed equally and then co-infiltrated into tobacco (*N. benthamiana*) leaves using an injection syringe. 48 h later, the leaves were collected (about 2 g of infiltrated leaves) and ground into good powder in liquid nitrogen for nuclear protein extraction. Samples were re-suspended in 25 mL CLB1 (50 mM HEPES, pH 7.5, 150 mM NaCl, 1 mM EDTA, 0.04% (v/v) β-mercaptoethanol, 1% (v/v) Triton X-100, 10% (v/v) glycerol, 1×Cocktail) and incubated at 4 °C for 30 min. The suspensions were first filtrated through a monolayer layer of Miracloth (Millipore) and then filtrated through a double layer of Miracloth, and centrifuged at 3000×*g* for 20 min at 4 °C. The pellets were washed twice with 1 mL CLB2 (50 mM HEPES, pH 7.5, 150 mM NaCl, 1 mM EDTA, 1% (v/v) Triton X-100, 10% (v/v) glycerol, 1 ×Cocktail) and re-suspended in 500 µL CLB2 added with 25 µL 5% (w/v) SDS. The suspensions were sonicated five times and centrifuged at 12,000×*g* for 10 min at 4 °C. Another 1.5 mL CLB2 buffer was added for resuspension. The nuclear proteins were incubated with 50 µL Anti-GFP mAb-Magnetic Beads (MBL) and incubated overnight with rotation at 4 °C. The beads were washed 5 times with washing buffer (50 mM HEPES, pH 7.5, 150 mM NaCl, 10% (v/v) glycerol, 0.1% (v/v) TritonX-100, 1 mM EDTA, 1 × Cocktail). The proteins were released by boiling at 100 °C for 10 min and subjected to Western blotting assays using anti-GFP (Abiocode; M0802-3a, 1:1000 dilution) and anti-FLAG (Sigma; F1804, 1:3000 dilution) antibodies. Blotting signals were detected by the PMCapture software (Version 1.00) of a Chemiluminescence Imaging System (Tanon 5500, Shanghai, China). Proteins were detected by western blots with anti-GFP or anti-FLAG antibodies. Values came from three biological replicates.

### Constructs and transgenic plants

The artificial microRNA was used to direct gene silencing^54^. For the *AtSCC3*RNAi vector construct, a 404-bp PCR product corresponding to the pre-miRNA stem-loop sequence of the *Arabidopsis* miR gene *miR319a* was amplified from genomic DNA and cloned into the vector *pCambia1300*. The vector was then used as a template for subsequent PCR amplification and replacement of the endogenous miR319a/miR319a* with amiRNA/amiRNA*^55^. The targets of *AtSCC3* were designed by a web-based tool (http://wmd.weigelworld.org). The primers are listed in Supplementary Data 8.

The full length of *AtSYN4* genomic DNA including the upstream regulatory sequence was subcloned into *Eco*RI/*Sal*I-treated vector *pCambia1300-N1-YFP* to generate *pSYN4-SYN4-YFP* construct. The *atsyn4* mutant was transformed with GV3101 harboring *pSYN4-SYN4-YFP* to generate *pSYN4-SYN4-YFP/atsyn4*. Primers used were shown in Supplementary Data 8.

The coding sequences of *AtSCC3* were subcloned into *Eco*RI/*Sal*I-treated vector *pCambia1300-35S-N1-YFP* to generate *Pro35S:AtSCC3-YFP*. *Arabidopsis* Col-0 plants were transformed with GV3101 harboring *Pro35S:AtSCC3-YFP* to generate the *AtSCC3-YFP*/Col-0 over-expression lines. Primers used were shown in Supplementary Data 8. All constructs were confirmed by sequencing and introduced into *Agrobacterium tumefaciens* strain GV3101 by electroporation. Transgenic *Arabidopsis* plants were generated by floral dipping^56^.

### Histone extraction and immunoblot assay

A total of 2 g of 7-day-old seedlings of Col-0, *atsyn4*, *atctf7*, *SCC3*RNAi-1, and *SCC3*RNAi-2 were harvested, and ground in liquid nitrogen into a good powder. The resulting powder was suspended in solution A containing 0.4 M sucrose, 10 mM Tris–HCl pH 8, 10 mM MgCl_2_, and 5 mM β-mercaptoethanol. The slurry was filtered through two layers of Miracloth (Millipore, 475855-1R) and the filtrate was centrifuged at 12,000×*g* for 10 min. The pelleted material was washed by solution B (0.25 M sucrose, 10 mM Tris–HCl pH 8, 10 mM MgCl_2_, 1% Triton X-100, and 5 mM β-mercaptoethanol) and centrifuged at 12,000×*g* for 10 min. Afterward, the pelleted chromatin was suspended in solution C (1.7 M sucrose, 10 mM Tris–HCl pH 8, 0.15% Triton X-100, 2 mM MgCl_2_, and 5 mM β-mercaptoethanol) and centrifuged at 27,000×*g* for 30 min. The extracted chromatin was then treated with 0.4 N H_2_SO_4_ overnight, and the proteins were precipitated with trichloroacetic acid (TCA) in a final concentration of 20% (w/v). The precipitated proteins were then washed three times with cold acetone, air-dried, suspended, and boiled for 10 min. Proteins were separated in 15% SDS–PAGE gels. Western blots were performed with anti-H2Aub1 (Cell Signaling 8240S, 1:2000 dilution) and anti-H3 (Sigma H0164, 1:3000 dilution) antibodies. Blotting signals were detected by the PMCapture software (Version 1.00) of a Chemiluminescence Imaging System (Tanon 5500, Shanghai, China).

### ChIP-seq and ChIP-qPCR assay

*Arabidopsis* Col-0 and *pSYN4-SYN4-YFP/atsyn4* plants were used for ChIP-seq. Plants were grown at 21 °C on MS agar plates supplemented with 1.5% sucrose and 0.8% agar. For ChIP assay^57^, 10 g fresh 7-day-old whole seedlings were cross-linked in the crosslink buffer (0.4 M sucrose, 10 mM Tris–HCl (pH 8.0), 1 mM PMSF, 1 mM EDTA, 1% formaldehyde) for 3 × 5 min using vacuum infiltration and the reaction was terminated in 2 M glycine. The extracted nuclei were immunoprecipitated with anti-CSCC3 (ABclonal WG-02026D), anti-GFP (Abcam ab290) antibodies (5 μg/2 g plant sample), and incubated at 4 °C overnight. After reverse cross-linking, DNA was extracted by phenol-chloroform method and sheared to an average size of 300 bp using a sonicator (Bioruptor, Diagenode). Sequencing library was constructed according to the standard Illumina protocol. For ChIP-seq, two immunoprecipitations from independent biological replicates were processed for next-generation sequencing.

For ChIP-qPCRs, anti-GFP (Abcam ab290), anti-H2Aub1 (Cell Signaling 8240S), and the AtSCC3-specific antibody (anti-CSCC3, ABclonal WG-02026D) generated by using 200 amino acids (898–1098) in AtSCC3 protein as the antigen was used. The fragments, 1-1, 1-2, 1-3 of AT1G64385 selected from chr1:23,895,129–23,909,562; 3-1, 3-2 of AT3G23440 selected from chr3:8,401,608–8,409,299; 4-1, 4-2, 4-3 of AT4G16370 and AT4G16380 selected from chr4:9,245,532–9,260,363 were tested by ChIP-qPCR assay. Con-1, con-3 and con-4 were negative controls from sequences chr1:23,745,058–23,745,114, chr3:8,401,561–8,401,814 and chr5: 9,378,482–9,378,752. Primers used for ChIP real-time PCR are listed in Supplementary Data 8. ChIP-qRCR data is collected by Bio-Rad CFX96.

### ChIP-seq data processing

Low-quality and adapter sequences were trimmed from the reads using cutadapt (v1.18) with parameters: -a AGATCGGAAGAGC -A AGATCGGAAGAGC–trim-n -m 50 -q 20, 20. Then, the reads were mapped to the *Arabidopsis thaliana* TAIR10 genome using Bowtie2 (v2.3.4.3) with parameters: -N 0--no-discordant --no-mixed --no-unal. SAMTools (v1.9) was used to transfer the mapping results from SAM format to position-sorted SAM format. Next, the duplicated reads were removed by markdup from sambamba (v0.6.8). The BAM files were then converted to BigWig files using bamCoverage from the deepTools suite (v3.1.3) with parameters: –normalizeUsing RPKM –binSize 25. The Integrative Genomics Viewer (IGV) was used to show the signal of AtSYN4/AtSCC3 enrichment in a certain genomic region in a track view. Peaks were called using MACS2 (v 2.1.1) callpeak with default parameters. Motif calling was performed using Homer. Peak locations and annotated genes of histone modifications (H3K27me3 and H2Aub1) were obtained from Zhou et al.^12^.

### Yeast one-hybrid assay

Yeast one-hybrid assays were performed according to the Matchmaker Yeast One-Hybrid System User Manual (PT1031-1 Clontech). Coding sequences of *AtSCC3* and *AtSYN4* were PCR-amplified and subcloned into pGADT7 (Clontech). The sequence1, sequence2, and sequence3 were 50 bp G-box (CACGTG) counting DNA selected from the promoter region of *AT1G64385*, *AT3G23440*, and *AT4G16380*, respectively. Mutant 1, mutant 2, and mutant 3 are mutations with the three sequences of G-box (CACGTG) in the promoters mutated into GGGGGG. The sequences were generated by primer annealing from primers synthesized, then subcloned into *Eco*RI/*Sac*I-treated vector *pHISi-1*. Resultant constructs were transformed into yeast strain YM4271. Yeast transformants were grown on synthetic dropout (-Leu/-His) medium containing 80 mM 3-AT for 3 days and observed. Primers used are listed in Supplementary Data 8.

### RNA extraction and qRT-PCR analysis

The *Arabidopsis* Col-0, *SCC3*RNAi-2, *atsyn4*, *atctf7*, and *atsyn2atsyn4* mutants were used for RNA-seq. Plants were grown at 21 °C in long-day conditions (16 h light and 8 h dark cycles) on Murashige & Skoog (MS) medium supplemented with 1.5% sucrose and 0.8% agar. The total RNAs were extracted from 7-day-old *Arabidopsis* seedlings using Trizol reagent (TIANGEN), and the libraries were constructed according to a standard protocol (Illumina). All of the data were from three biological replicates.

For each sample, RNA (about 2 µg) was used as a template for reverse transcription using ReverTra Ace qPCR RT Master Mix with gDNA Remove Kit (Toyobo) according to the manufacturer’s instructions. qRT-PCRs were conducted in a total volume of 20 μL containing 10 μL SYBR Premix Ex-Taq, 0.2 μg cDNA, primers (0.2 mM), and 8.3 μL double distilled water. *ACTIN2* (At3g18780) was used for data normalization. Primers used for qRT-PCRs were listed in Supplementary Data 8. Values came from three biological replicates each with three technical repeats. qRT-RCR data was collected by Bio-Rad CFX96.

### RNA-seq data analysis

Low-quality and adapter sequences were trimmed using cutadapt (v1.18) with the parameters: -a AGATCGGAAGAGC -A AGATCGGAAGAGC–trim-n -m 50 -q 20, 20. RNA sequencing reads were aligned using HISAT2 (v2.1.0) to the *Arabidopsis thaliana* TAIR10 genome. Mapped reads were subsequently assembled into transcripts using featureCounts (v1.6.1) with parameters: -p -C -B to calculate read counts under TAIR10 genome reference. Three independent biological replicates were performed. Data for *Atbmi1a/b/c* RNA-seq analysis were previously published^12,13^.

### Reporting summary

Further information on research design is available in the Nature Portfolio Reporting Summary linked to this article.

### Supplementary information

Supplementary Information

Description of Additional Supplementary Files

Supplementary Data 1

Supplementary Data 2

Supplementary Data 3

Supplementary Data 4

Supplementary Data 5

Supplementary Data 6

Supplementary Data 7

Supplementary Data 8

Reporting Summary

### Source data

Source Data

## Data Availability

The AtSYN4-ChIP, AtSCC3-ChIP, Input-ChIP, and RNA-seq datasets have been deposited to NCBI under accession PRJNA681034. Source data are provided with this paper.
