# Author Correction: Histone H2A monoubiquitination marks are targeted to specific sites by cohesin subunits in *Arabidopsis*

**DOI:** 10.1038/s41467-024-48977-9

**Published:** 2024-06-05

**Authors:** Yu Zhang, Min Ma, Meng Liu, Aiqing Sun, Xiaoyun Zheng, Kunpeng Liu, Chunmei Yin, Chuanshun Li, Cizhong Jiang, Xiaoyu Tu, Yuda Fang

**Affiliations:** 1 Joint Center for Single Cell Biology, School of Agriculture and Biology, Shanghai Jiao Tong University, 200240 Shanghai, China; 2 Key Laboratory of Spine and Spinal Cord Injury Repair and Regeneration of Ministry of Education, Orthopaedic Department of Tongji Hospital, School of Life Sciences and Technology, Tongji University, 200065 Shanghai, China

**Keywords:** Plant molecular biology, Epigenomics, Epigenetics, Plant genetics

Correction to: *Nature Communications* 10.1038/s41467-023-36788-3, published online 3 March 2023

In the Acknowledgements section of this article the grant number relating to the National Science Foundation of China given for Yuda Fang was incorrectly given as 3217040101 and should have been 32170585. The original article has been corrected.

