# Supplementary material for: Histone H2A monoubiquitination marks are targeted to specific sites by cohesin subunits in Arabidopsis
Source: Nat Commun. 2023 Mar 3;14:1209. doi: 10.1038/s41467-023-36788-3 (PMC9984397; doi:10.1038/s41467-023-36788-3)
Supplement: Supplementary file 1 — Supplementary Information [file 41467_2023_36788_MOESM1_ESM.pdf]

Histone H2A monoubiquitination marks are targeted to specific sites by cohesin subunits in *Arabidopsis*

Zhang *et al.*

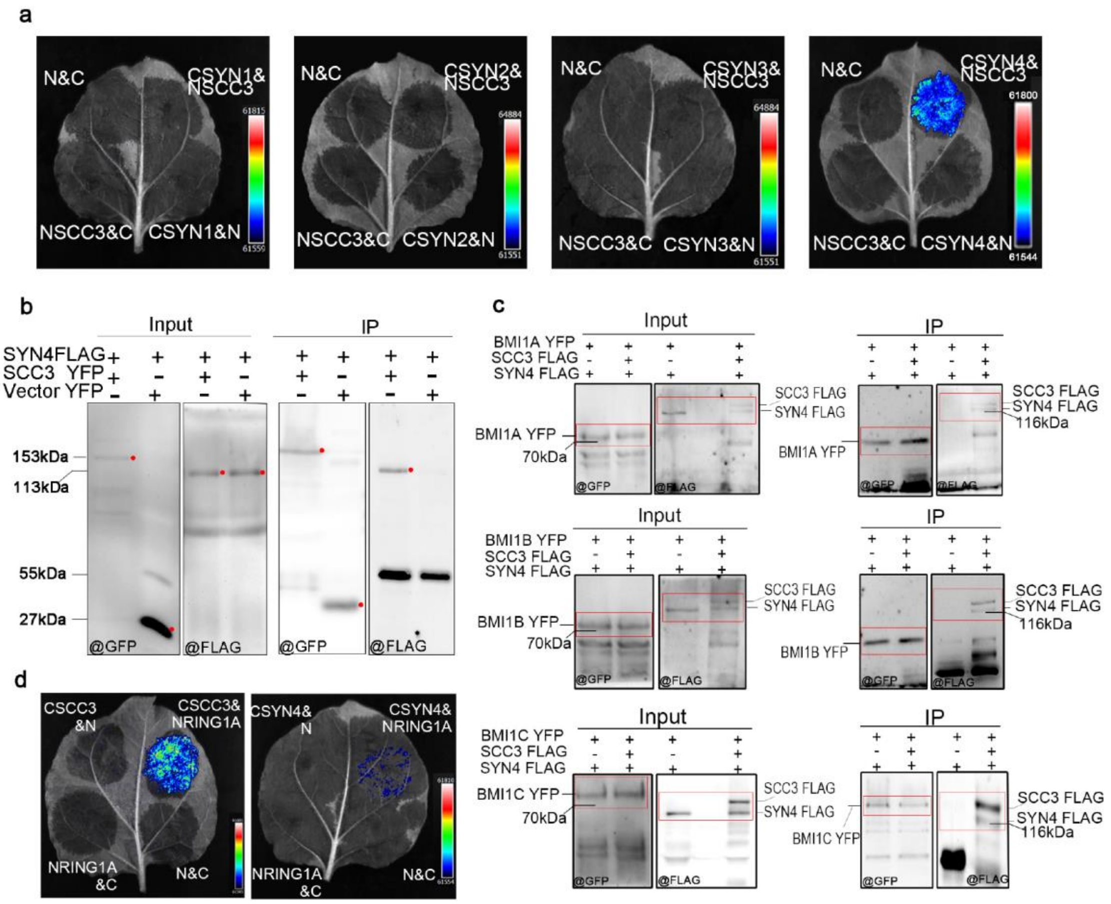

Supplementary Figure 1. AtSYN4 interacts with AtSCC3 and AtRING1A. a LUC complementation assays indicated that AtSCC3 interacts with AtSYN4, but not AtSYN1-3. **b** Co-IP assays showing the interaction between AtSYN4 and AtSCC3. The molecular weights of SCC3-YFP and SYN4-FLAG recombinant proteins are 152.7 kDa and 116.7 kDa, respectively. Red dots on the right of bands indicate the expected sizes of expressed proteins. The experiment was repeated independently three times with similar results. **c** Co-IP experiments verified that AtSYN4 can interact with AtBMI1A/B/C only when AtSCC3 is expressed. The cropped areas marked by red box in each blots correspond to the micrographs in Figure 1c. **d** LUC complementation imaging assays indicated that AtRING1A interacts with AtSCC3 and AtSYN4. The CSCC3 (AtSCC3 fused to the C-terminal fragment of LUC) or CSYN4 (AtSYN4 fused to the C-terminal fragment of LUC) was co-expressed with NRING1A (RING1A fused to the N-terminal fragment of LUC) in the tobacco leaves and the fluorescence signals were visualized 48 h after inoculation. The experiment was repeated independently three times with similar results. The unprocessed scans of the blot images are provided as a Source Data file.

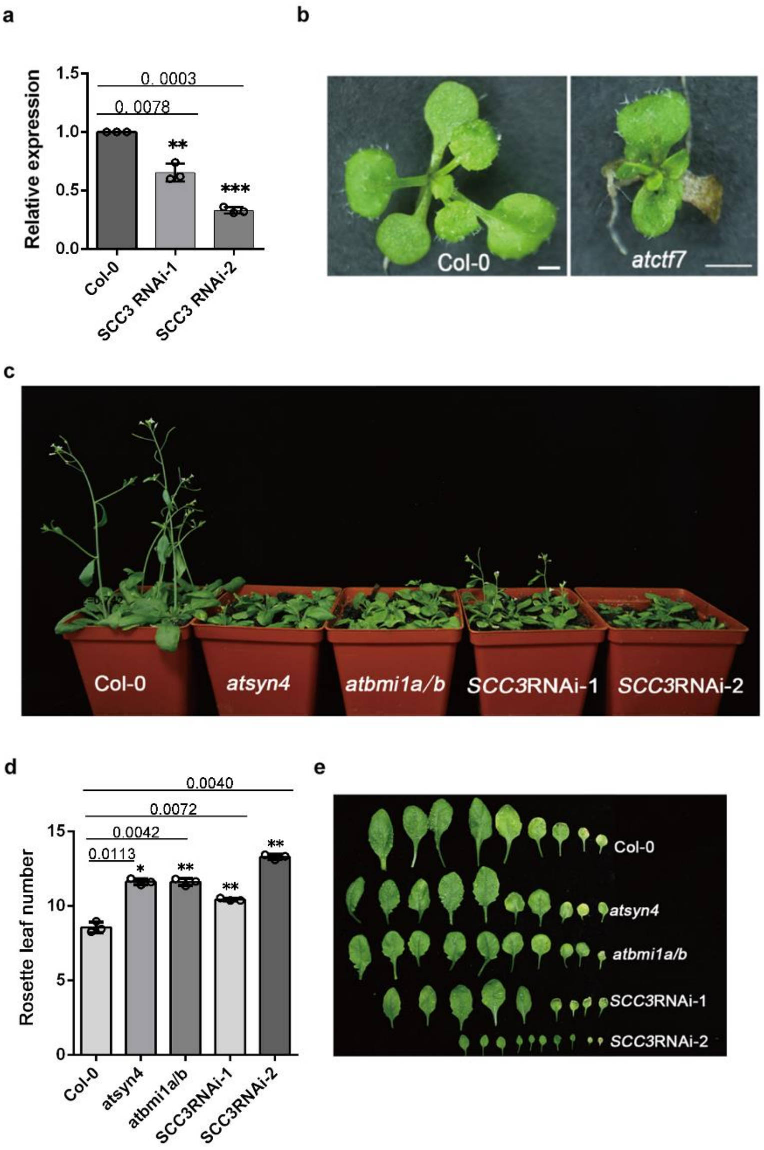

Supplementary Figure 2. Phenotypic comparisons among *atbmi1a/b*, *atsyn4* and *AtSCC3* RNAi lines. a Transcript levels of *AtSCC3* in Col-0 and two independent *AtSCC3*RNAi knock-down lines, *SCC3*RNAi-1 and *SCC3*RNAi-2; Data are mean ± SD of three biological replicates. Statistical analysis was performed with one-tailed Student's *t* tests; *p* value："***"<0.001, "**"＜0.01, and the exact *p* values are noted . The raw data of the bar graphs are provided as a Source Data file. **b** Visual phenotypes of 14-day-old Col-0 and *atctf7* plant. The *atctf7* mutant develops abnormally. Scale bar=1 mm. **c** Visual phenotypes of 25-day-old Col-0, *atsyn4*, *atbmi1a/b*, *SCC3*RNAi-1 and *SCC3*RNAi-2 under long days. **d** Statistical analysis of rosette serrate numbers of Col-0, *atsyn4*, *atbmi1a/b*, *SCC3*RNAi-1 and *SCC3*RNAi-2 upon bolting. Data are mean ± SD of three biological repeats (n=50). Statistical analysis was performed with two-tailed Student's *t* tests; *p* value："**"＜0.01, "*"＜0.1, and the exact *p* values are noted . The raw data of the bar graphs are provided as a Source Data file. **e** Serrated rosette leaves of 25-day-old Col-0, *atsyn4*, *atbmi1a/b*, *SCC3*RNAi-1 and *SCC3*RNAi-2 under long days.

a b

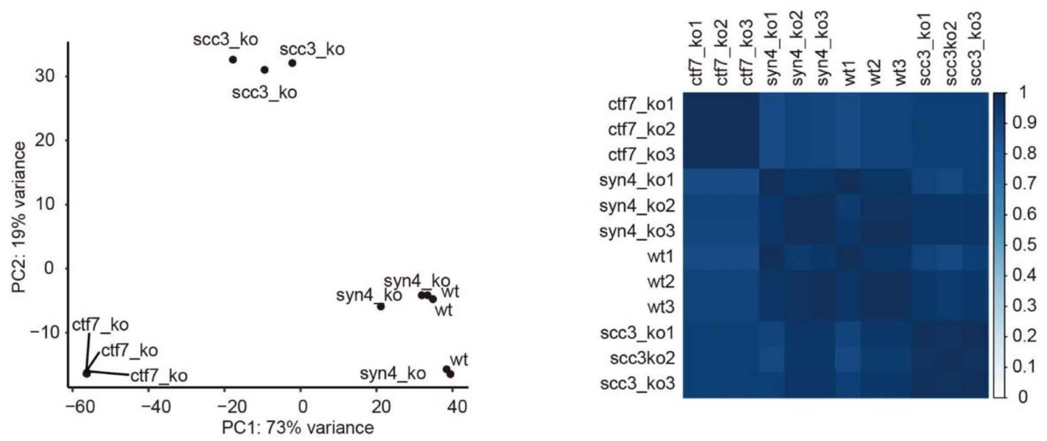

Supplementary Figure 3. Quality control of RNA-seq experiments. a PCA plot of gene transcriptions in RNA-seq data of wild type (wt), *AtSCC3* RNAi-2, *atsyn4* and *atctf7* mutants. **b** Heatmap shows clustering gene expression profile of the biological replicates in **a**.

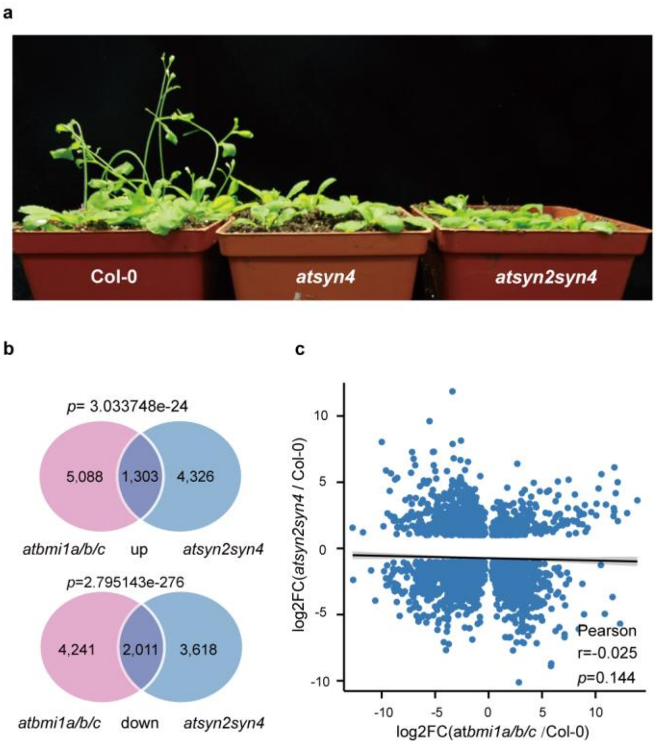

Supplementary Figure 4. Transcriptome comparisons between *atbmi1a/b/c* and *atsyn2atsyn4* mutant plants. a Visual phenotypes of 25-day-old Col-0, *atsyn4*, *atsyn2atsyn4* under long days. **b** Venn diagrams showing overlaps of the up- or down-regulated genes between *atsyn2atsyn4* and *atbmi1a/b/c* mutants. Significance was examined by the hypergeometric test. **c** Scatter plots showing correlations of the up- and down-regulated genes between *atsyn2atsyn4* and *atbmi1a/b/c* mutants. Statistical analysis was performed with two-tailed Student's *t* tests. $|\log_2$fold change$| > 1$ and *p* adjusted < 0.05. The values of log2FC(*atbmi1a/b/c*/Col-0) and log2FC(*atsyn2atsyn4* /Col-0) are provided as a source data file.

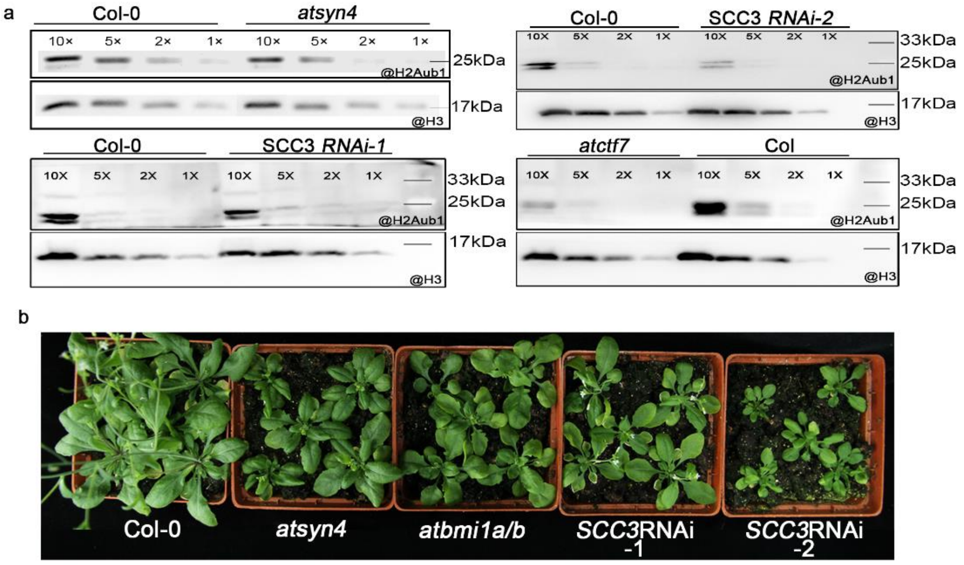

Supplementary Figure 5. H2Aub1 levels in *atsyn4*, *SCC3*RNAi-1, *SCC3*RNAi-2 and *atctf7* mutants. a Western blot analysis of H2Aub1 levels in the histone enriched extracts from Col-0, *atsyn4*, *SCC3*RNAi-1, *SCC3*RNAi-2 and *atctf7* plants. The experiment was repeated independently three times with similar results. The unprocessed scans of the blot images are provided as a Source Data file. **b** Top views of 25-day-old Col-0, *atsyn4, atbmi1a/b*, *SCC3*RNAi-1 and *SCC3*RNAi-2 under long days.

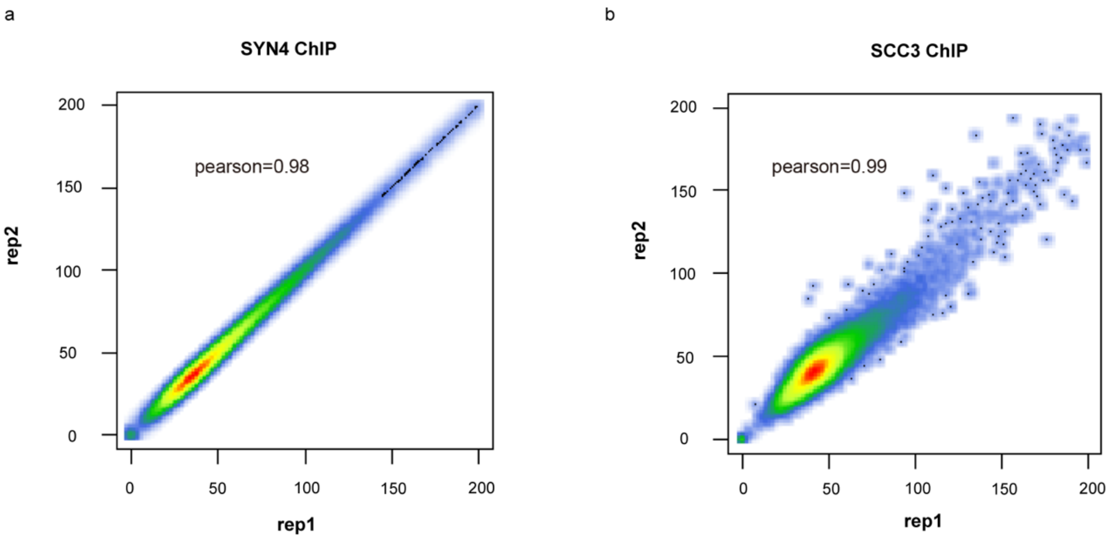

Supplementary Figure 6. Quality control of ChIP-seq experiments. a Correlations between two biological replicates of AtSYN4 ChIP-seq data. **b** Correlations between two biological replicates of AtSCC3 ChIP-seq data.

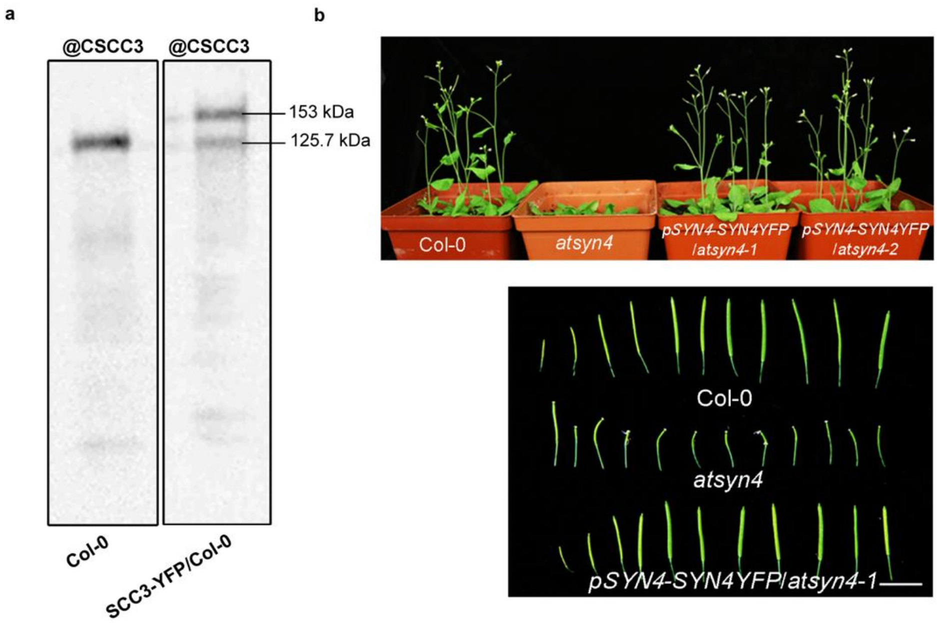

Supplementary Figure 7. Validation of CSCC3 antibody and *AtSYN4* transgenic plants. a Western blot assay confirmed the specificity and sensitivity of CSCC3 antibody. Total proteins were extracted from Col-0 and *AtSCC3-YFP/* Col-0 plants. The experiment was repeated independently three times with similar results. The unprocessed scans of the blot images are provided as a Source Data file. **b** Two independent genetic complementary lines (*pSYN4-SYN4-YFP*/*atsyn4-1* and *pSYN4-SYN4-YFP*/*atsyn4-2*) restore the late flowering and sterility phenotypes of the *atsyn4* mutant. Scale bar=20 mm.

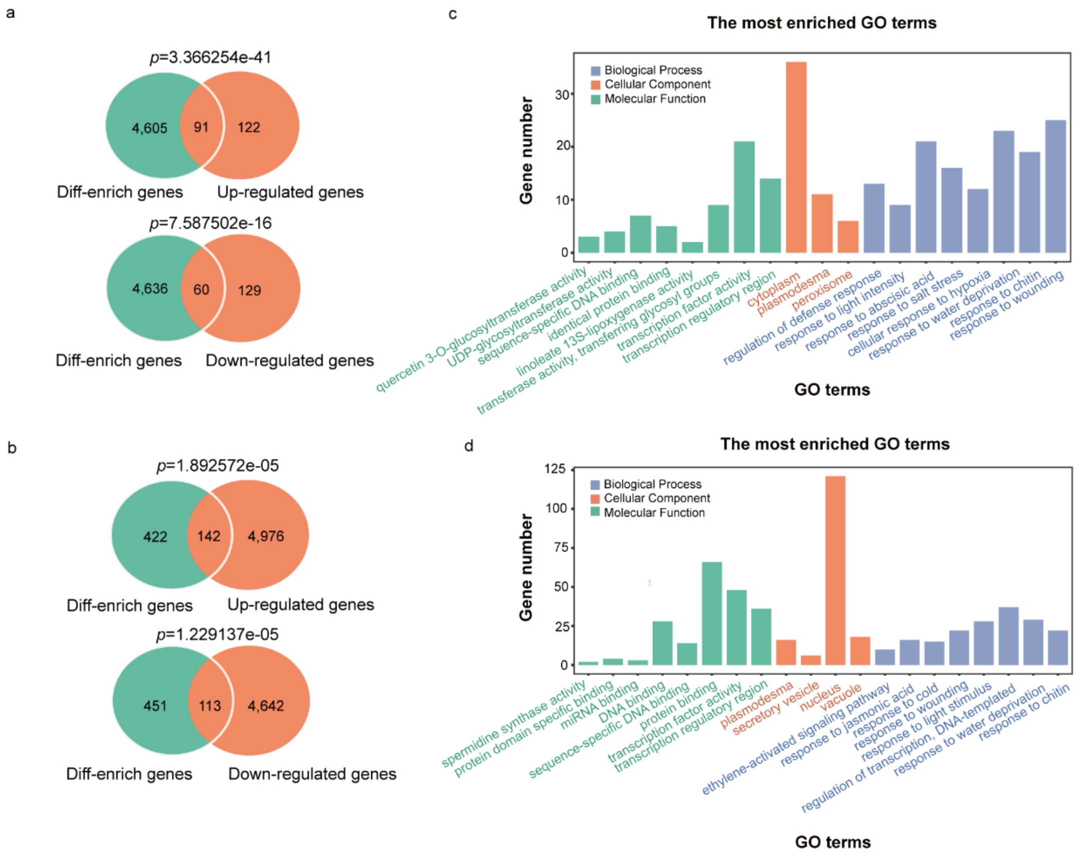

Supplementary Figure 8. AtSYN4 and AtSCC3 regulate the expression of genes targeted by H2Aub1. a Venn diagrams showing the overlaps between up- or down-regulated genes in *atsyn4* and AtSYN4-differentially enriched genes targeted by H2Aub1 (Diff-enrich genes). Significance was examined by the hypergeometric test. **b** Venn diagrams showing the overlaps between up- or down-regulated genes in *SCC3*RNAi-2 and AtSCC3-differentially enriched genes targeted by H2Aub1 (Diff-enrich genes). Significance was examined by the hypergeometric test. **c** Enrichment analysis of partial significantly enriched GO terms were performed on the overlapped differential genes in **a**. The abscissa represents different GO terms that are significantly enriched (p value < 0.05), and the ordinate is the number of genes enriched on different GO terms. **d** Enrichment analysis of partial significantly enriched GO terms were performed on the overlapped differential genes in **c**. The abscissa represents different GO terms that are significantly enriched (p value < 0.05), and the ordinate is the number of genes enriched on different GO terms.
