# Supplementary material for: Histone H2A monoubiquitination marks are targeted to specific sites by cohesin subunits in Arabidopsis
Source: Nat Commun. 2023 Mar 3;14:1209. doi: 10.1038/s41467-023-36788-3 (PMC9984397; doi:10.1038/s41467-023-36788-3)
Supplement: Supplementary file 2 — Description of Additional Supplementary Files [file 41467_2023_36788_MOESM2_ESM.pdf]

Description of Additional Supplementary Files

File Name: Supplementary Data 1
Description: Differentially expressed genes in SCC3RNAi-2 line.

File Name: Supplementary Data 2
Description: Differentially expressed genes in atbmi1a/b/c line.

File Name: Supplementary Data 3
Description: Differentially expressed genes in atsyn4 line.

File Name: Supplementary Data 4
Description: Differentially expressed genes in atsyn2atsyn4 double mutant.

File Name: Supplementary Data 5
Description: Differentially expressed genes in atctf7 line.

File Name: Supplementary Data 6
Description: AtSCC3 ChIP-seq peaks.

File Name: Supplementary Data 7
Description: AtSYN4 ChIP-seq peaks.

File Name: Supplementary Data 8
Description: List of primers used in this study.
