# Supplementary material for: Histone H2A monoubiquitination marks are targeted to specific sites by cohesin subunits in Arabidopsis
Source: Nat Commun. 2023 Mar 3;14:1209. doi: 10.1038/s41467-023-36788-3 (PMC9984397; doi:10.1038/s41467-023-36788-3)
Supplement: Supplementary file 11 — Reporting Summary [file 41467_2023_36788_MOESM11_ESM.pdf]

nature portfolio

Corresponding author(s): Yuda Fang, Xiaoyu Tu, Cizhong Jiang

Last updated by author(s): Feb 12, 2023

Reporting Summary

Nature Portfolio wishes to improve the reproducibility of the work that we publish. This form provides structure for consistency and transparency in reporting. For further information on Nature Portfolio policies, see our Editorial Policies and the Editorial Policy Checklist.

Statistics

For all statistical analyses, confirm that the following items are present in the figure legend, table legend, main text, or Methods section.

n/a	Confirmed	
☐	☒	The exact sample size (*n*) for each experimental group/condition, given as a discrete number and unit of measurement
☐	☒	A statement on whether measurements were taken from distinct samples or whether the same sample was measured repeatedly
☐	☒	The statistical test(s) used AND whether they are one- or two-sided<br>*Only common tests should be described solely by name; describe more complex techniques in the Methods section.*
☒	☐	A description of all covariates tested
☒	☐	A description of any assumptions or corrections, such as tests of normality and adjustment for multiple comparisons
☐	☒	A full description of the statistical parameters including central tendency (e.g. means) or other basic estimates (e.g. regression coefficient) AND variation (e.g. standard deviation) or associated estimates of uncertainty (e.g. confidence intervals)
☐	☒	For null hypothesis testing, the test statistic (e.g. *F*, *t*, *r*) with confidence intervals, effect sizes, degrees of freedom and *P* value noted<br>*Give P values as exact values whenever suitable.*
☒	☐	For Bayesian analysis, information on the choice of priors and Markov chain Monte Carlo settings
☒	☐	For hierarchical and complex designs, identification of the appropriate level for tests and full reporting of outcomes
☒	☐	Estimates of effect sizes (e.g. Cohen's *d*, Pearson's *r*), indicating how they were calculated

Our web collection on statistics for biologists contains articles on many of the points above.

Software and code

Policy information about availability of computer code

Data collection	(1)ChIP-seq libraries and RNA-seq libraries were prepared and sequenced according to the standard Illumina protocol. Part of the ChiP-Seq data(H2Aub1 and H3K27me) were downloaded from the NCBI citing the accessiom numbers provided by published papers through the software sratoolkit (v2.10.0), with parameters: prefetch --option-file sra.txt and fastq-dump --split-3.<br>Reference: Zhou, Y., Romero-Campero, F. J., Gomez-Zambrano, A., Turck, F. & Calonje, M. H2A monoubiquitination in Arabidopsis thaliana is generally independent of LHP1 and PRC2 activity. Genome Biol 18, 69 (2017).<br>(2)The luciferase signals and the blotting signals were detected by the PMCapture software (Version 1.00) of a Chemiluminescence Imaging System (Tanon 5500, Shanghai, China).
Data analysis	Open source software used include: (1) ChIP-seq : cutadapt (v1.18) with parameters: -a AGATCGGAAGAGC -A AGATCGGAAGAGC --trim-n -m 50 -q 20, 20; Bowtie2 (v2.3.4.3) with parameters: -N 0 --no-discordant --no-mixed --no-unal. samtools (v1.9); sambamba (v0.6.8); bamCoverage from the deeptools suite (v3.1.3) with parameters: --normalize using RPKM --binSize 25; MACS2 (v 2.1.1) callpeak with default parameters. (2) RNA-seq: cutadapt (v1.18) with the parameters: -a AGATCGGAAGAGC -A AGATCGGAAGAGC --trim-n -m 50 -q 20, 20; HISAT2 (v2.1.0) with the parameters:hisat2-build -p 4 and hisait2 -p 16; featureCounts (v1.6.1) with parameters: -p -C -B.

For manuscripts utilizing custom algorithms or software that are central to the research but not yet described in published literature, software must be made available to editors and reviewers. We strongly encourage code deposition in a community repository (e.g. GitHub). See the Nature Portfolio guidelines for submitting code & software for further information.

Data

Policy information about availability of data

All manuscripts must include a data availability statement. This statement should provide the following information, where applicable:

- Accession codes, unique identifiers, or web links for publicly available datasets
- A description of any restrictions on data availability
- For clinical datasets or third party data, please ensure that the statement adheres to our policy

Data supporting the findings of this work are available within the paper and its supplementary Information files. A reporting summary for this article is available as a supplementary Information file. The datasets generated and analyzed during the current study are available from the corresponding author upon request. The SYN4-ChIP, SCC3-ChIP, Input-ChIP and RNA-seq raw data have been deposited in NCBI (PRJNA681034), and can be downloaded from https://www.ncbi.nlm.nih.gov/bioproject/PRJNA681034. The final processed data of ChIP-seq have been deposited in GEO via GEO Series accession number GSE205736.

Human research participants

Policy information about studies involving human research participants and Sex and Gender in Research.

Reporting on sex and gender	N/A
Population characteristics	N/A
Recruitment	N/A
Ethics oversight	N/A

Note that full information on the approval of the study protocol must also be provided in the manuscript.

Field-specific reporting

Please select the one below that is the best fit for your research. If you are not sure, read the appropriate sections before making your selection.

☒ Life sciences ☐ Behavioural & social sciences ☐ Ecological, evolutionary & environmental sciences

For a reference copy of the document with all sections, see nature.com/documents/nr-reporting-summary-flat.pdf

Life sciences study design

All studies must disclose on these points even when the disclosure is negative.

Sample size	No sample-size calculations were performed. Sample size was determined to be adequate based on the experimental purpose, accuracy requirements and the feasibility of practical operation and that can reflect the consistency of measurements.<br>For RNA-Seq and ChIP-Seq, 7-day-old seedlings of Col-0 were used. For phenotype analysis, after stratification, the seedlings were grown in soil in long day condition (16 h light and 8 h dark cycles), and the days of flowering and rosette numbers were scored with three biological replicates.
Data exclusions	No data were excluded.
Replication	The days of flowering and rosette numbers were scored with three biological replicates. Yeast two-hybrid interaction assays, firefly luciferase (LUC) complementation imaging assay, Co-Immunoprecipitation (Co-IP) assay were performed with three biological replicates. For ChIP-seq, two immunoprecipitations from independent biological replicates were processed for next-generation sequencing library preparation. qRT-PCR analysis and RNA-seq were performed with three biological replicates.
Randomization	All genotypes were grown side by side and randomly placed in the chamber.<br>Plant materials were randomly picked up for our data collection and analyses.
Blinding	No blinding was used. There was no subjective bias in this study.

Reporting for specific materials, systems and methods

We require information from authors about some types of materials, experimental systems and methods used in many studies. Here, indicate whether each material, system or method listed is relevant to your study. If you are not sure if a list item applies to your research, read the appropriate section before selecting a response.

Materials & experimental systems

n/a	Involved in the study
☐	☒ Antibodies
☒	☐ Eukaryotic cell lines
☒	☐ Palaeontology and archaeology
☒	☐ Animals and other organisms
☒	☐ Clinical data
☒	☐ Dual use research of concern

Methods

n/a	Involved in the study
☐	☒ ChIP-seq
☒	☐ Flow cytometry
☒	☐ MRI-based neuroimaging

Antibodies

Antibodies used	1 We used 898-1098 amino acids of the AtSCC3 protein as an antigen to generate an AtSCC3-specific antibody: anti-CSCC3 (Abclonal, catalog number:WG-02026D).<br>2 GFP antibody for AtSYN4-ChIP-seq: anti-GFP (Abcam #ab290).<br>3 Anti-H3 (Sigma, H0164)<br>4 Anti-GFP (Abiocode, M0802-3a)<br>5 Anti-FLAG (Sigma, F1804)<br>6 Anti-H2Aub1(CST,8240S)
Validation	Western blot assay confirmed the specificity and sensitivity of CSCC3 antibody, and the experimental results are shown in the supplemental figure 6a. Total proteins were extracted from Col-0 and SCC3-YFP/ Col-0 plants. The other antibodies have been validated by the company using western plot or indirect immunofluorescence.

ChIP-seq

Data deposition

☒ Confirm that both raw and final processed data have been deposited in a public database such as GEO.

☒ Confirm that you have deposited or provided access to graph files (e.g. BED files) for the called peaks.

Data access links<br>*May remain private before publication.*	1 The raw data have been deposited in https://www.ncbi.nlm.nih.gov/bioproject/PRJNA681034<br>2 The final processed data have been deposited in https://www.ncbi.nlm.nih.gov/geo/query/acc.cgi?acc=GSE205736.
Files in database submission	col_20180601N_GACTAG_S29_L003_R1_001.fastq.gz<br>col_20180601N_GACTAG_S29_L003_R2_001.fastq.gz<br>wt2_R1.fq.gz<br>wt2_R2.fq.gz<br>wt3_R1.fq.gz<br>wt3_R2.fq.gz<br>syn4_ko_rep1.R1.fq.gz<br>syn4_ko_rep1.R2.fq.gz<br>syn4_ko_rep2.R1.fq.gz<br>syn4_ko_rep2.R2.fq.gz<br>syn4_ko_rep3.R1.fq.gz<br>syn4_ko_rep3.R2.fq.gz<br>syn2syn4-RNAseq-biological replicate1<br>syn2syn4-RNAseq-biological replicate2<br>syn2syn4-RNAseq-biological replicate3<br>scc3_RNAi-1_FRAS202462584-1r_1.clean.fq.gz<br>scc3_RNAi-1_FRAS202462584-1r_2.clean.fq.gz<br>scc3_RNAi-2_FRAS202462585-1r_1.clean.fq.gz<br>scc3_RNAi-2_FRAS202462585-1r_2.clean.fq.gz<br>scc3_RNAi-3_FRAS202462586-1r_1.clean.fq.gz<br>scc3_RNAi-3_FRAS202462586-1r_2.clean.fq.gz<br>ctf7_1_1.fq.gz<br>ctf7_1_2.fq.gz<br>ctf7_2_1.fq.gz<br>ctf7_2_2.fq.gz<br>ctf7_3_1.fq.gz<br>ctf7_3_2.fq.gz<br>chip-SYN4-1_RRC03961_1.fq.gz<br>chip-SYN4-1_RRC03961_2.fq.gz<br>chip-SYN4-2_RRC03962_1.fq.gz<br>chip-SYN4-2_RRC03962_2.fq.gz

chip-SCC3-1_RRC03964_1.fq.gz
chip-SCC3-1_RRC03964_2.fq.gz
chip-SCC3-2_RRC03965_1.fq.gz
chip-SCC3-2_RRC03965_2.fq.gz
input-ChIP-rep1_RRC03963_1.clean.fq.gz
input-ChIP-rep1_RRC03963_2.clean.fq.gz
input-ChIP-rep2_RRC03833_1.fq.gz
input-ChIP-rep2_RRC03833_2.fq.gz
SYN4_1.bw
SYN4_2.bw
SCC3_1.bw
SCC3_2.bw
input_1.bw
input_2.bw

Genome browser session (e.g. UCSC)

Tracks for all ChIP-Seq data generated in this study can be found in https://www.ncbi.nlm.nih.gov/geo/query/acc.cgi?acc=GSE205736

Methodology

Replicates

For ChIP-seq, two immunoprecipitations from independent biological replicates were processed for next-generation sequencing library preparation.

Sequencing depth

Sample name Total number of reads length of reads SE/PE
input-ChIP-rep1 22201040 300 PE
Input-chIP-rep2 20721843 300 PE
SYN4-ChIP-biological replicate 1 23756114 300 PE
SYN4-ChIP-biological replicate 2 30987341 300 PE
SCC3-ChIP-biological replicate1 28474271 300 PE
SCC3-ChIP-biological replicate2 23571398 300 PE

Antibodies

1.We used 898-1098 amino acids of the AtSCC3 protein as an antigen to generate an AtSCC3-specific antibody: anti-CSCC3 (Abclonal, lot number:WG-02026D).
2 GFP antibody for AtSYN4-ChIP-seq: anti-GFP (Abcam #ab290).

Peak calling parameters

The reads were mapped to the Arabidopsis thaliana TAIR10 genome using Bowtie2 (v2.3.4.3) with parameters: -N 0 --no-discordant --no-mixed --no-unal. Peaks were called using macs2 (v 2.1.1) callpeak with default parameters.

Data quality

Peak calling result file Number of peaks with FDR<5% and Fold enrichment>1(our method) Number of peaks with FDR<5% and Fold enrichment>5
SCC3_peaks.narrowPeak 1978 5
SNY4_peaks.narrowPeak 11224 40

Software

ChIP-seq: cutadapt (v1.18) with parameters: -a AGATCGGAAGAGC -A AGATCGGAAGAGC --trim-n -m 50 -q 20, 20; Bowtie2 (v2.3.4.3) with parameters: -N 0 --no-discordant --no-mixed --no-unal. Samtools (v1.9); sambamba (v0.6.8); bamCoverage from the deeptools suite (v3.1.3) with parameters: --normalize using RPKM --binSize 25; MACS2 (v 2.1.1) callpeak with default parameters.
